# Association of Light Exposure on Physical Activity and Sedentary Time in Young People

**DOI:** 10.3390/ijerph120302941

**Published:** 2015-03-10

**Authors:** Daniel Aggio, Lee Smith, Abigail Fisher, Mark Hamer

**Affiliations:** 1 Health Behaviour Research Centre, University College London, London WC1E6BT, UK; E-Mails: lee.smith@ucl.ac.uk (L.S.); abigail.fisher@ucl.ac.uk (A.F.); 2 Physical Activity Research Group, Department of Epidemiology and Public Health, University College London, London WC1E6BT, UK; E-Mail: m.hamer@ucl.ac.uk

**Keywords:** Accelerometry, sitting time, adolescents, children, outdoors

## Abstract

*Background*: To investigate whether light exposure was associated with objectively measured physical activity (PA) and sedentary behaviour in young people. *Methods*: Participants (n = 229, 46.7% female) were young people (mean 8.8 years [SD ± 2.2]) from the borough of Camden, UK. Daily sedentary time, moderate and vigorous PA (MVPA) and light exposure were measured using a tri-axial accelerometer with an ambient light sensor during the summer. Multiple linear regression models examined associations between average daily light exposure, sedentary time and time in MVPA. Models were repeated investigating weekdays and weekend days separately. Analyses were adjusted for pre-specified covariables, including age, sex, device wear time, ethnic group, school and body fat. *Results*: There were significant associations between average daily light exposure and time sedentary (β coefficient *=* −11.2, 95% CI, −19.0 to −3.4) and in MVPA (β coefficient = 3.5, 95% CI, 1.2 to 5.9). Light exposure was significantly associated with weekend sedentary time (β coefficient *=* −10.0, 95% CI, −17.6, −2.4), weekend MVPA (β coefficient *=* 3.7, 95% CI, 1.7, 5.7), weekday sedentary time (β coefficient *=* −15.0, 95% CI, −22.7 to −7.2), but not weekday MVPA (β coefficient *=* 2.0, 95% CI, −0.5 to 4.5). *Conclusion*: Average daily light exposure is positively associated with time in MVPA and negatively associated with sedentary time. Increasing daylight exposure may be a useful intervention strategy for promoting physical activity.

## 1. Introduction

Regular participation in physical activity is associated with a reduced risk of several non-communicable disease risk factors in young people (aged 4 to 15 years) [1,2]. Despite this, less than a quarter of young people in England are sufficiently active to meet the current recommendations [3] of 60 min moderate-to-vigorous intensity physical activity (MVPA) per day. Moreover, prolonged periods of sedentary behaviour (e.g. sitting) have been found to be associated with several cardiometabolic risk factors, independent of physical activity levels in this age group [4].

Evidence has been accumulating for a role of the physical environment in promoting or hindering physical activity behaviour in young people [5]. For example, factors such as access to green/open spaces [6,7] and walking/cycling infrastructures are associated with higher physical activity levels. One environmental factor that has received little attention so far is light exposure. More light exposure has been found to be associated with several health outcomes, including lower adiposity [8], reduced levels of depression [9], reduced risk of type 2 diabetes mellitus [10] and some cancers [11]. In addition, seasonal patterns in clinical events have been observed, with better survival rates in the summer [12,13]. These health outcomes are strongly associated with physical activity levels [14,15,16], but the relationship between light exposure and physical activity is poorly understood. 

Existing data suggest young people are consistently more active when outside [17,18,19]. Cleland *et al.* reported that every additional hour per day spent outdoors results in an extra 27 min week^−1^ MVPA among older girls (aged 10–12 years), and an extra 20 min∙week^−1^ MVPA among older boys [18]. In addition, time spent outdoors has been found to be correlated with overweight [18] and independent mobility [20]. It is plausible to assume that greater time spent outdoors is associated with more light exposure. Therefore, it could be hypothesised that a high light exposure is negatively associated with sedentary time and positively associated with MVPA. To our knowledge there are no studies investigating the associations between objective light exposure, physical activity and sedentary time in young people. The aim of this study was to investigate whether light exposure was associated with objectively measured physical activity and sedentary behaviour in a sample of young people residing in central London.

## 2. Methods

Participants were recruited as part of the Camden Active Spaces project. Full details on study participants and procedures have previously been reported [21]. In brief, participants (n = 319, 42% female) were young people (aged 5 to 15 years) from six schools (5 primary and 1 secondary) from the London borough of Camden, UK. Data were collected in term time between June and July 2014. Head teachers provided written consent for all young people to take part. Parental consent was obtained using an opt-out approach. Participant assent was also attained in secondary school children. Finally, all participants had the option of opting out of any or all of the measures being taken at the point of testing. On the day of testing, stature was measured to the nearest 0.1 cm using a Leicester Height Measure. Mass and body composition were measured using a Tanita SC-330 Body Composition Analyser (Tanita Inc, Illinois, IL, USA). Participants also self-reported age, gender and ethnicity with the assistance of the study team. Once anthropometric measures were completed, participants were instructed to wear a tri-axial accelerometer with an ambient light sensor (wGT3X-BT model, ActiGraph Ltd, FL, USA) on the right hip for 7 consecutive days during waking hours, but not during water-based activities or sleep. Participants were fitted with the device over their clothing and were instructed to keep the device over clothing throughout the 7 days where possible. After 7 days the devices were collected by the research team. Ethical approval was granted by the University College London Research Ethics Committee (4400/002).

### 2.1. Exposure Measure: Light Exposure

The Actigraph tri-axial accelerometer wGT3X-BT model has an ambient light sensor that gives lux data that can be integrated with the activity data. Lux is a measure of the intensity of light. A lux value of >10,000 indicates exposure to full daylight and values up to 500 lux typically indicate room lighting [22].

In this study, the average daily lux value was used as a measure of light exposure throughout the day (between 07:00 and 00:00). Average daily lux values are underreported in the literature, but one study found in a sample of adults that only 1 hour per day is spent above 1000 lux [8], indicating that average daily lux values will range a lot lower. Average daily lux was positively skewed and thus it was log transformed prior to analysis.

### 2.2. Outcome Measures: Physical Activity and Sedentary Time

The tri-axial accelerometers provide an objective measure of physical activity and sedentary time and are considered valid and reliable for measuring these behaviours in young people [23,24,25]. Once downloaded, data were exported and processed in excel. The first day (a partial wear day) of data was excluded from analyses because of the wide range of times at which participants received their accelerometer. Total physical activity, time spent sedentary and in MVPA (min/day) were derived using age-specific cut points as used previously [26]. Specifically, minutes with less than 100 accelerometer counts per minute (cpm) [27] were deemed as sedentary and minutes with more than 3000 cpm as time in MVPA [27,28,29]. Bouts of 60 minutes or more of continuous zero counts were excluded from the data and considered as non-wear time [30]. The inclusion criteria required one day of at least 500 minutes wear time between 07:00 and 00:00, which mirrors previously used criteria in the largest accelerometer-based study in children [26]. Using all valid days, a daily average for time sedentary and in MVPA was calculated in minutes per day. Physical activity and light exposure may differ at the weekend when compared to weekdays. On the weekend children generally have more discretionary time and therefore greater opportunity to roam and play outdoors. Therefore daily averages were also calculated for weekdays and weekend days separately using only valid weekdays and weekend days, respectively.

### 2.3. Covariates

Participants’ age, sex, ethnic group and school were included as covariates. Body fat and total time spent wearing the accelerometer (min/day), determined by the inclusion criteria described above, were also included in the models. Final analyses also mutually controlled for MVPA and sedentary time in the sedentary and MVPA models, respectively.

### 2.4. Statistical Analysis

Differences in characteristics between boys and girls were examined using t-tests (continuous variables) and chi-squared tests (categorical variables). The association between light exposure (log lux) and average time spent per day sedentary and in MVPA were examined using multiple linear regression models. The initial models examined the association between light exposure and time sedentary and in MVPA while adjusting for age, sex and wear time. Further models were performed adjusting for school, ethnic group and body fat. We also included sedentary time as a covariate in the MVPA model and MVPA as a covariate in the sedentary time model. All models were then repeated for weekdays and weekend days separately. We tested for statistical interactions with respect to sex although none were found. Thus boys and girls were pooled together and the analyses were adjusted for sex. All analyses were conducted using SPSS version 22.

## 3. Results

From the initial sample of 319, a total of 269 participants (84%) provided at least 1 day of valid wear time. The final sample reduced to 229 participants (46.7% female) due to missing covariable data. Descriptive characteristics of the study sample can be found in Table 1. In the final analytic sample, all participants provided at least one valid day of accelerometer wear time, with 60.7% providing at least five valid days and just 7.4% providing only one valid day (mean device wear time was 4.7 days [SD ± 1.7]). Mean age of the sample was 8.8 years (SD ± 2.3), and 36.2% were White British. Activity levels (total and MVPA) were higher in boys than girls. Boys also had lower sedentary time. There were no significant differences in light exposure and device wear time between boys and girls. Average daily light exposure ranged from 0 to 735 lux.

**Table 1:** Descriptive characteristics of participants stratified by sex.

	Boys (n = 122)	Girls (n = 107)
Age (years)	8.6 ± 2.3	9.1 ± 2.2
Body fat percentage	21.0 ± 7.5	23.4 ± 8.1 *
Ethnic group (% white British)	36.1	36.4
Total Physical Activity (min/day)	411.9 ± 75.0	388.3 ± 70.5 *
MVPA (min/day)	34.5 ± 18.5	22.3 ± 13.0 **
Sedentary (min/day)	338.2 ± 80.5	369.8 ± 99.5 **
Average Daily Light exposure (raw lux value) ^†^	114.5 ± 155	116.3 ± 197
Device wear time (min/day)	750.1 ± 94.2	758.1 ± 89.8

Data are presented as mean (SD). Moderate-to-vigorous intensity physical activity (MVPA) ^†^ Lux data presented as median and interquartile range. * Significant difference (*p* < 0.05), ** (*p* < 0.01).

Table 2 presents the associations between light exposure and time spent sedentary and in MVPA. There were significant associations between light exposure and time spent sedentary and in MVPA, which were only marginally attenuated in the final adjusted models. We further examined associations for just weekdays and weekends. For this sub analysis, 228 and 157 participants had valid accelerometry data for weekdays and weekends, respectively, accompanied by complete lux, self-reported and body composition data. Characteristics did not differ from those included in the full analysis. Light exposure was significantly associated with weekend sedentary time and MVPA. It was also associated with weekday sedentary time, but not with weekday MVPA in the final adjusted models. All associations persisted after removing subjects with 0 lux values (see supplementary table).

**Table 2:** Association between average daily, weekday and weekend light exposure (Log Lux) and time spent sedentary and in MVPA (min/day).

	Model 1	Model 2
	B coefficient ^†^ (95% CI)	B coefficien t ^†^ (95% CI)
MVPA		
Daily (n = 229)	5.2 (3.0, 7.5) **	3.5 (1.2, 5.9) **
Weekday (n = 228)	4.2 (1.9, 6.5) **	2.0 (−0.5, 4.5)
Weekend (n = 157)	4.9 (3.0, 6.7) **	3.7 (1.7, 5.7) **
Sedentary time		
Daily (n = 229)	−13.9 (−21.3, −6.4) **	−11.2 (−19.0, −3.4) **
Weekday (n = 228)	−16.3 (−23.8, −8.7) **	−15.0 (−22.7, −7.2) **
Weekend (n = 157)	−13.9 (−21.0, −6.8) **	−10.0 (−17.6, −2.4) *

** *p* < 0.01, * *p* < 0.05; **^†^** coefficient reflects minutes/day of MVPA or sedentary time per unit increase in LUX; *Model 1* adjusted for age, sex and device wear time. *Model 2* additionally adjusted for ethnic group, school, body fat and daily MVPA or daily sedentary time.

## 4. Discussion

This paper aimed to investigate whether light exposure was associated with objectively measured physical activity and sedentary behaviour. Previous research has suggested that greater light exposure is associated with more favourable health outcomes in adults [8,9,10,11]. One study found a positive trend between light exposure and physical activity, although this was only in a small sample of adults [31]. The present analyses show in young people that light exposure is associated with more time in MVPA and less time sedentary, after controlling for covariates. Interestingly, the independent nature of these associations suggests that light exposure may be associated with sedentary behaviours and physical activity separately and that they are not simply displacing each other. In the weekday final model, there was no association between light exposure and time in MVPA, which was in contrast to weekend days. This may be explained by limited opportunities to be outside and active during the school day compared to the weekend when young people are likely to have more freedom to be active outside.

To the best of our knowledge, this study is the first to investigate objectively measured light exposure and its relationship with both physical activity and sedentary time in young people. It would seem intuitive that children with higher light exposure have spent more time outside. Previous research has found that young people are consistently more active when outside [4,5,8]. Thus, a plausible explanation for the results found in this paper may be that young people who had higher light exposure spent more time outdoors and consequently spent more time in MVPA and less time sedentary. Furthermore, existing research has shown seasonal variation in physical activity levels in children. Months with higher daylight hours (spring and summer) are associated with higher physical activity levels compared to months with lower daylight hours (autumn and winter) in young people [32,33,34]. Exposure to daylight may therefore be an important determinant of physical activity levels in young people. 

Strategies to increase daylight exposure may increase activity levels by encouraging more outdoor activity. In support, a recent study found that longer evening daylight was associated with increases in daily physical activity [35]. The introduction of additional daylight saving measures could yield worthwhile public health benefits. Other strategies may encourage children to spend more time outdoors, such as by providing them with a safe outdoor arena to play in or by implementing classroom activities outside during the summer months. 

Strengths of this paper include the ethnically diverse sample and objectively measured light exposure, physical activity and sedentary behaviour. One potential limitation of this study is the lack of evidence on validity of the light sensor in free-living conditions. Further, it remains to be seen whether a reliable cut point can be determined for defining indoor and outdoor time for use in epidemiological research. A potential issue with using the Actigraph light sensor to determine time spent outdoors is that some crossover exists between the thresholds for defining indoor and outdoor light, making classification of outdoors/indoor time difficult. For example, lux values on a very dark overcast day may be comparable with indoor office lighting on the same day. Nevertheless, all data were collected over a 6 week period during the summer when outside levels of light were relatively stable. Also, taking an average daily lux value, as presented in this study, should attenuate this problem but does not allow for minute-by-minute analysis. Various other techniques have been used to quantify time spent outdoors, with comparable limitations. For example, GPS has been used in this way but, similarly, evidence for its validity is lacking. Furthermore, GPS may misclassify time spent outdoors because signals can still sometimes be received when inside. Since the Actigraph device was worn around the hip, light exposure may have been underestimated if the light sensor was covered by clothing, which may explain some of the low average daily lux values. More research is required to validate this as a measure of time spent outdoors, but there are potential advantages of using the integrated Actigraph light sensor in this way over other objective measures of time spent outdoors. Like all cross sectional studies we cannot infer causality from the data, so cannot interpret if light might cause younger people to be active or if active children are incidentally also exposed to more light because the outdoor environment is more conducive to activity. It is important for future research to determine the effects of increasing light exposure on physical activity and sedentary behaviours.

## 5. Conclusions

Light exposure was positively associated with time spent in MVPA and negatively associated with sedentary time in young people. Light exposure was also associated with weekend MVPA and sedentary time, weekday sedentary time, but not weekday MVPA. Increasing daylight exposure may be a useful intervention strategy for promoting physical activity.

## Supplementary Files

Supplementary File 1
