# Supplementary material for: Association of Light Exposure on Physical Activity and Sedentary Time in Young People
Source: Int J Environ Res Public Health. 2015 Mar 10;12(3):2941–9. doi: 10.3390/ijerph120302941 (PMC4377944; doi:10.3390/ijerph120302941)
Supplement: Supplementary File 1 [file ijerph-12-02941-s001.pdf]

Supplementary Information

Association of Light Exposure on Physical Activity and Sedentary Time in Young People

Table S1. Association between average daily, weekday and weekend light exposure (Log Lux) and time spent sedentary and in MVPA (min/day).

	**Model 1**<br>**B Coefficient † (95% CI)**	**Model 2**<br>**B Coefficient † (95% CI)**
MVPA		
Daily (n = 228)	5.3 (3.0, 7.6) **	3.6 (1.2, 6.0) **
Weekday (n = 226)	4.1 (1.7, 6.5) **	1.8 (−0.7, 4.4)
Weekend (n = 153)	4.8 (2.7, 6.8) **	3.7 (1.6, 5.9) **
Sedentary time		
Daily (n = 228)	−13.6 (−21.2, −6.0) **	−11.0 (−18.9, −3.0) **
Weekday (n = 226)	−15.8 (−23.7, −8.0) **	−14.8 (−22.9, −6.7) **
Weekend (n = 153)	−12.1 (−19.5, −4.8) **	−8.4 (−16.2, −0.5)*

Subjects with lux vales <1 were excluded from this analysis. ** $p < 0.01$, * $p < 0.05$; † coefficient reflects minutes/day of MVPA or sedentary time per unit increase in LUX; *Model 1* adjusted for age, sex and device wear time. *Model 2* additionally adjusted for ethnic group, school, body fat and daily MVPA or daily sedentary time.

© 2015 by the authors; licensee MDPI, Basel, Switzerland. This article is an open access article distributed under the terms and conditions of the Creative Commons Attribution license (http://creativecommons.org/licenses/by/4.0/).
